# Complex agro-ecosystems for food security in a changing climate

**DOI:** 10.1002/ece3.271

**Published:** 2012-07

**Authors:** Uma Khumairoh, Jeroen CJ Groot, Egbert A Lantinga

**Affiliations:** 1 Biological Farming Systems Group, Wageningen Centre for Agro-ecology and Systems Analysis (WaCASA), Wageningen UniversityP.O. Box 563, 6700 AN Wageningen, The Netherlands; 2 Integrated Organic Farming System Research Centre (IORC), Faculty of Agriculture, Brawijaya UniversityJl. Veteran 65145 Malang, Indonesia; 3 Department for Sustainable Management of Resources, Radboud University NijmegenP.O. Box 9010, 6500 GL Nijmegen, The Netherlands

**Keywords:** Agro-ecology, nutrient cycling, pest suppression, rice, smallholders

## Abstract

Attempts to increase food crop yields by intensifying agricultural systems using high inputs of nonrenewable resources and chemicals frequently lead to de-gradation of natural resources, whereas most technological innovations are not accessible for smallholders that represent the majority of farmers world wide. Alternatively, cocultures consisting of assemblages of plant and animal species can support ecological processes of nutrient cycling and pest control, which may lead to increasing yields and declining susceptibility to extreme weather conditions with increasing complexity of the systems. Here we show that enhancing the complexity of a rice production system by adding combinations of compost, azolla, ducks, and fish resulted in strongly increased grain yields and revenues in a season with extremely adverse weather conditions on East Java, Indonesia. We found that azolla, duck, and fish increased plant nutrient content, tillering and leaf area expansion, and strongly reduced the density of six different pests. In the most complex system comprising all components the highest grain yield was obtained. The net revenues of this system from sales of rice grain, fish, and ducks, after correction for extra costs, were 114% higher than rice cultivation with only compost as fertilizer. These results provide more insight in the agro-ecological processes and demonstrate how complex agricultural systems can contribute to food security in a changing climate. If smallholders can be trained to manage these systems and are supported for initial investments by credits, their livelihoods can be improved while producing in an ecologically benign way.

## Introduction

Narrowing existing gaps between actual and potential crop yields has been identified as one of the priorities to secure food availability for the increasing global human population (Tilman et al. 2002; IAASTD 2009; Graham-Rowe 2011). Currently, the actual yields obtained by farmers are ca. 50% of the potential yields, and in exceptional cases in irrigated crops up to 80% is reached (Lobell et al. 2009). These yield gaps are caused by insufficient or unbalanced supply of water and nutrients, damage due to weeds, pest and diseases, and losses caused by weather-related events such as extreme temperatures, severe rainfall events, and prolonged periods of drought (Van Ittersum and Rabbinge 1997; Lobell et al. 2009). In the near future, farmers and their agricultural systems should be able to deal with even stronger fluctuations in environmental and weather conditions, since climate change is expected to increase the incidence of weather anomalies (Naylor et al. 2007; Lobell et al. 2009; Meinke et al. 2009; Gornall et al. 2010).

Increasing both productivity and robustness of agricultural systems will require concerted developments in technological innovation and improved crop cultivation techniques (Uphoff 2010; Graham-Rowe 2011). However, attempts to increase food crop yields by intensifying agricultural systems using high inputs of nonrenewable resources and chemicals frequently lead to degradation of soils and other natural resources (Matson et al. 1997; Steinfeld et al. 2006; IAASTD 2009), whereas most technological innovations are not accessible for smallholders that represent the majority of farmers world wide (Kiers et al. 2008; Herrero et al. 2010). Thus, in many cases, increasing and stabilizing the productivity of food crops will primarily depend on better management of crops, ecological processes and organic resources to increase soil structure and fertility, and to protect crops against weed competition and damage by pests and diseases.

Complex agricultural systems consisting of assemblages of plant and animal species can support ecological processes of nutrient cycling and pest control, which may lead to higher yields and reduced susceptibility to extreme weather conditions when the complexity of the systems increases (Van Noordwijk and Swift 1999; Altieri 2002; Shennan 2008). However, there is a lack of scientific knowledge concerning agro-ecological processes of plant growth and development, effectiveness of pest suppression, nutrient cycling, and the productive performance of agricultural systems on gradients of complexity and under extreme conditions. Integrated rice cultivation can be considered as a pertinent model system for the analysis of ecological processes in complex agro-ecosystems, due to its importance as a food crop and the inherent complexity of cultivation practices involving soil, water, and plant resources.

We conducted a field trial to investigate the attainable yields in complex agro-ecosystems by studying the combined effects of integration of compost application, ducks, fish, and azolla in a flooded rice system in a season with extremely adverse weather conditions of high rainfall on East Java, Indonesia. Rice was cultivated using selected practices from the System of Rice Intensification (SRI), that is larger planting distances (30 × 30 cm) of individual young (10-days old in the 2–3 leaf stage) seedlings and using compost as organic fertilizer (Kassam et al. 2011), but in contrast to SRI recommendations the fields were flooded to allow fish and duck integration. Ducks and fish are widely used in flooded rice production systems in Asia (Jana and Jana 2003; Lu and Li 2006; Cheng-fang et al. 2008), but less frequently applied jointly. More complex combinations involving also compost and azolla are scarce in practice and in scientific research, as only one production dataset (Cagauan et al. 2000) without further information on nutrient and pest dynamics is available. In these systems, compost can serve as a source of organic matter and nutrients to improve soil structure and crop nutrient status. This includes a sufficient provision of micronutrients that are frequently deficient when inorganic fertilizers are used (Kassam et al. 2011). Azolla is a floating fern that lives in symbiosis with the blue green algae *Anabaena azollae*, which can fix atmospheric nitrogen. Ducks and fish can feed on azolla, weeds, and pest organisms, thus improving nutrient cycling within the system and suppressing weed and pest populations (Cagauan et al. 2000; Jana and Jana 2003). The results obtained in the experiment reported here, with systems in a gradient of complexity, provide more insight in the agro-ecological processes and demonstrate how complex agricultural systems can contribute to food security in a changing climate.

## Methods

### General

The on-farm field experiment was conducted between 1 September and 12 December 2010, in Pagelaran, Malang district, East Java, Indonesia (8°10´27´´S, 112°35´58´´E), at an altitude of 335 m.a.s.l. on a clay-loam soil. The experimental layout was a randomized block design with two replicate blocks. The control treatment consisted of rice only (R), the other eight treatments consisted of combinations of nutrient management (compost (+C) and compost and azolla (+C+A)) and pest management (ducks (+D) and ducks and fish (+D+F)). This resulted in nine treatment combinations with a gradient in complexity. Experimental plots of 200 m^2^ were surrounded by dikes to prevent flows of nutrients, fish, and azolla between plots. Plots with ducks were fenced to prevent movement of ducks between plots and contained a duck house (1.5 m × 1.5 m × 1.0 m) constructed of bamboo and rice straw. Compost was produced of straw, duckweed, water hyacinth, and duck manure, heaped for a month and turned once. The compost was spread manually using a hoe.

Rice (*Oryza sativa*, cv. Ciherang) suitable for both wet and dry seasons was cultivated. The rice cultivar used is tolerant to brown plant hopper biotypes 2 and 3, as well as rice blast diseases strain III and IV. Plants were seeded at a rate of 20 kg/ha. In agreement with the recommendations of Kassam et al. (2011) to enhance the development of the root system, seedlings were transplanted 10 days after germination in the 2–3 leaf stage, and planted on hills with a planting distance of 30 × 30 cm, resulting in a plant density of ca. 11 plants m^–2^ (22–25 August 2010). Plots with azolla treatment were inoculated with a mixture of *Azolla pinnata* and *A. microphylla* at a rate of 2000 kg/ha. The azolla growth was stimulated by 28 kg/ha of SP-36 phosphorous fertilizer (Ca(H_2_PO_4_)_2_, with 5% of sulfur, and total P_2_O_5_ of 36%). Local ducks (*Anas platyrhynchos Javanicus*; local name: Mojosari) were introduced in the plots with duck treatment at a rate of 400 per hectare. Supplementary feed was supplied to the ducks at a rate of 75–150 g per animal per day, depending on the body weight. Nile tilapia (*Oreochromis niloticus*) of 10 cm length (ca. 30 g) was used in the fish treatments at a rate of 5000 per hectare. Azolla, ducks, and fish were released into plots 2 weeks after transplanting (8 September 2010). A second inoculation of azolla was performed on 15 September 2010 at the same rate as for the initial inoculation. Plots were weeded manually three times in plots without ducks and two times in plots with ducks.

### Measurements

Five plants per plot were harvested diagonally at early tillering (28 days after transplanting, DAT), maximum tillering (49 DAT), flowering (70 DAT), and grain filling (84 DAT). The final harvest was conducted at 112 DAT. The leaves were dissected of the plant and leaf area was measured using a Leaf Area Meter (Li310, LICOR Inc., Lincoln, Nebraska, USA). Leaves, stems, grains, and roots were oven-dried at 70°C for 72 h and subsequently weighed to determine the dry matter (DM) content. Samples were ground to pass a 1 mm screen in a hammer mill and analyzed for nutrient contents. Ducks and fish were removed at flowering (70 DAT) and weighed.

Snails (*Bellamya javanica*) were counted in an area of 30 cm^2^ around a rice plant for five plants per plot on a diagonal. Mature snails could be collected manually whereas smaller snails were recovered from soil. A square wooden box was used to mark the area, and then soil and snails were taken out by a shovel. The sample was sieved to collect the snails. Dead snails and eggs were not counted.

Yellow traps were used to measure insect populations of rice whorl maggots (*Nephotettix virescens*), leaf hoppers (*N. virescens*), plant hoppers (*Nilaparvata lugens*), and stem borers (*Scirpophaga incertulas* and *S. innotata*). Five yellow traps per plot located on a diagonal within the plot were tied on a bamboo stick, and then the bamboo stick was plugged onto a single rice hill in the morning. The population of rice bugs (*Leptocorisa oratorius*) was estimated by trapping in dead crabs attached to a bamboo stick (five traps per plot on a diagonal). The traps remained in the field for 24 h.

Total inputs and outputs of nitrogen, phosphorus, and potassium were calculated to discern the effects of nutrient additions to the system from effects of improved nutrient cycling within the system. The inputs included rice seeds, compost (consisting of duckweed, straw, and duck manure), duck feed (consisting of rice bran, corn, and dried fish), ducklings, juvenile fish, and phosphorus fertilizer; the composition of these inputs is presented in Table S1. In addition, for nitrogen we estimated atmospheric deposition of 0.4 g N m^–2^ and symbiotic fixation by azolla of 1.8 g N m^–2^. The outputs of nutrients were in rice grain, duck, and fish. Nutrient losses by leaching, volatilization, or denitrification were not included in the balance calculations, since we aimed to assess the contribution of improved nutrient cycling to the productive potential of the systems along the complexity gradient.

Costs for all inputs (seed, duck feed, manure, fish, and ducklings) and labor (for rice management, keeping ducks, and fish), equipment and water management were recorded. Revenues were calculated from all sold products: rice grain, ducks, and fish.

### Statistical analysis

The normal distribution of data was tested using skewness and kurtosis tests, where necessary log transformation was performed. Trends in annual average temperature and cumulative precipitation were tested with linear regression. Experimental treatment effects were tested by analysis of variance, and Tukey's post hoc test was used to establish significant differences between treatments. All statistical tests were conducted with SPSS 18 software package (SPSS Inc., Chicago, Illinois, USA).

## Results

**Figure 1:**
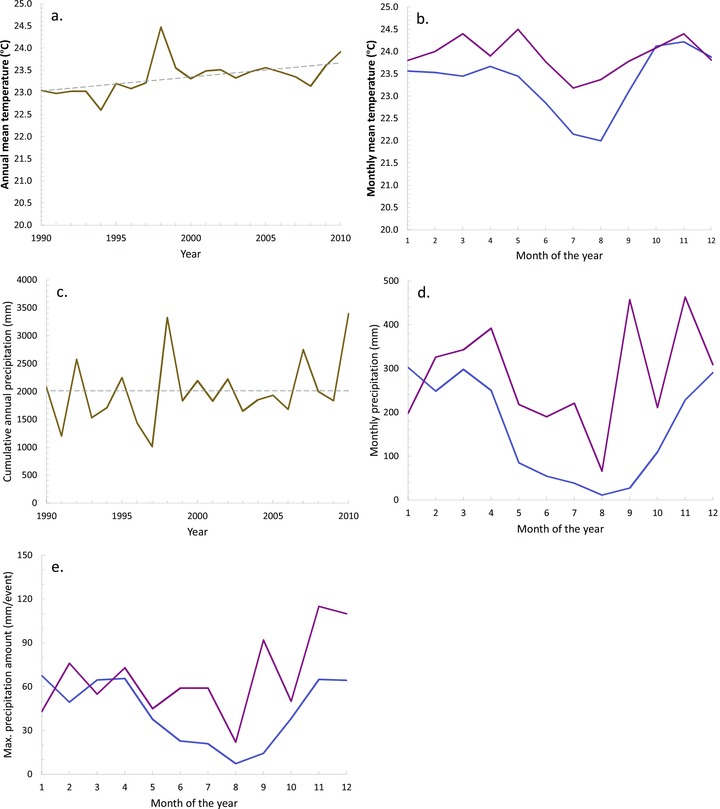
Trends in climatic conditions in the Malang region, Indonesia. (a) Average annual temperature. (b) Monthly mean temperature. (c) Cumulative annual precipitation. (d) Monthly amount of precipitation. (e) Maximum amount of precipitation in one event per month. Blue lines denote 20 years average (1990–2010), red lines represent the observations for the year of the experiment (2010), results of linear regressions are indicated with dashed lines.

Throughout the last 20 years the mean temperatures have significantly increased in the Malang district (*F*_1,19_ = 6.6848, *P* = 0.01814), but during the experiment the temperatures were not higher than the long-term average (Fig. 1a and b). In contrast, although annual precipitation rates have not shown an increasing trend in the period 1990–2010 (*F*_1,19_ = 1.8686, *P* = 0.1876; Fig. 1c), the total rainfall amounts in the years 1998 and 2010 have been extremes in terms of total rainfall and amounts per event (Fig. 1d and e), which resulted in undesired natural flooding and conditions that were very conducive for growth of pest populations in the trial presented here.

**Figure 2:**
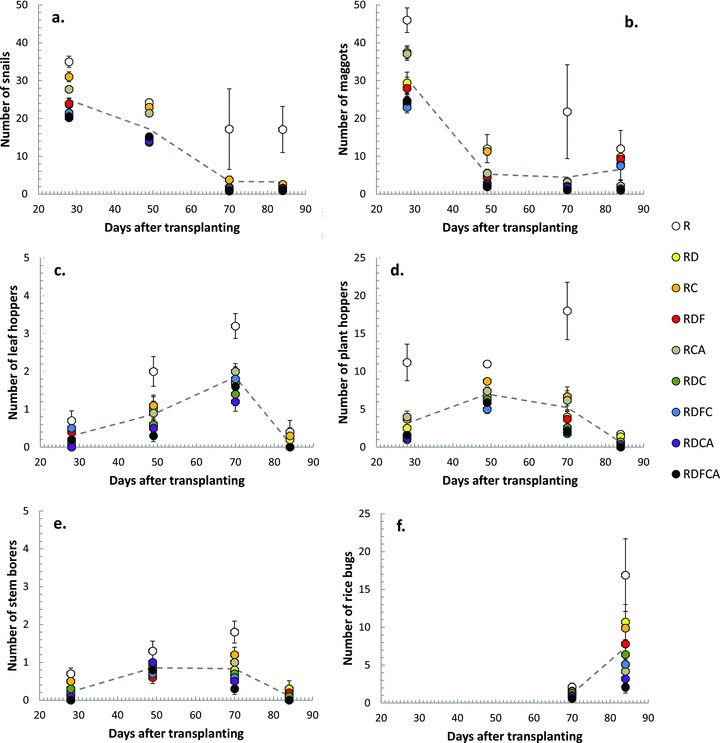
Abundance of plant pests per hill (± standard error of mean), (a) snails (*Bellamya javanica*), (b) rice whorl maggots (*Nephotettix virescens*), (c) leaf hoppers (*N. virescens*), (d) plant hoppers (*Nilaparvata lugens*), (e) stem borers (*Scirpophaga incertulas* and *S. innotata*), (f) rice bugs (*Leptocorisa oratorius*). R, rice; D, with ducks; C, with compost; F, with fish; A, with azolla. The dashed line indicates the trend in the overall average. Error bars represent standard error of the mean (*n* = 10).

The populations of six pest species were monitored (Fig. 2). Snails (*B. javanica*) and rice whorl maggots (*N. virescens*) were abundant directly after transplanting the seedlings but their populations declined throughout the experiment. Populations of leaf and plant hoppers (*N. virescens* and *N. lugens*) and stem borers (*S. incertulas* and *S. innotata*) were initially small but increased until flowering (ca. 60 DAT) and declined thereafter, whereas the number of rice bugs (*L. oratorius*) increased only from 70 DAT onwards, during grain filling. For all pests the size of the population was largest for the rice-only treatment, and lower for the more complex combinations of system production factors. In particular, the presence of ducks reduced the pest abundance. The rice plants in the more complex treatment combinations had higher leaf expansion rates and reduced plant stress as indicated by the lower values for biomass DM content and specific leaf area (Rubia-Sanchez et al. 1999; Quentin et al. 2010) (Fig. S1).

**Figure 3:**
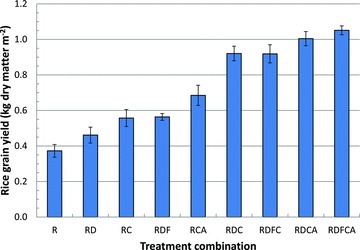
Yield of rice grain for increasingly complex rice cultivation systems (± standard error of mean). R = rice; D = with ducks; C = with compost; F = with fish; A = with azolla. Error bars represent standard error of the mean (*n* = 10).

The total biomass and grain yield increased with larger system complexity (*F*_8,81_ = 31.645, *P* < 0.001) and hence the highest grain yield of 1.06 kg DM m^–2^ was obtained in the most complex system comprising compost, azolla, ducks, and fish (Figs. 3 and S2). The higher grain yields for more complex combinations of production factors could be attributed to higher number of tillers per plant and larger seed weight due to application of compost and introduction of ducks, whereas the presence of azolla resulted in an increase of the number of grains per panicle (Table S2). The contribution of fish to grain yield was not significant (interaction between nutrient and pest protection treatments, *F*_4,81_ = 3.003, *P* = 0.023), but it resulted in significantly higher nitrogen and potassium accumulation in the rice crop (Table S3).

Analysis of the relation between total nutrient inputs and total nutrient outputs allowed to discriminate the effects of adding nutrients with increasing complexity (for instance in compost and duck feed) from impacts of ducks and fish on nutrient cycling processes. This analysis revealed that both ducks and fish improved nitrogen cycling, only ducks contributed to potassium cycling and that ducks and fish did not affect the phosphorus cycling in the various rice cultivation systems (Fig. S3).

**Figure 4:**
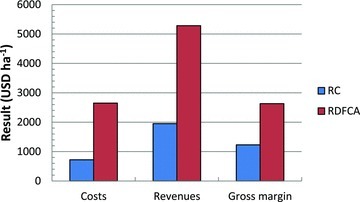
Economic results of rice cultivation in terms of costs, revenues and gross margin for rice with compost (RC) and a complex system involving ducks, fish, compost, and azolla (RDFCA), expressed in United States Dollars (USD) per hectare.

When compared to the generally applied organic rice cultivation practices where compost is applied, the more complex systems required extra investments to purchase the applied production factors of young ducks and fish (Table S4). However, these investments were more than compensated by the additional revenues from the sales of the better yielding rice grain and the mature ducks and fish, as demonstrated from the comparison between the rice with compost (RC) treatment and the most complex treatment combination (Fig. 4).

## Discussion

Increasing potential and actual cereal yields has been identified as one of the main challenges to secure food supply to a large part of the global population (Cassman et al. 2003; Normile 2008; Godfray et al. 2010). Compared to other food crops, for rice there is less pressure on developments in plant breeding and genetic engineering to improve productivity, because the seed market is limited as most of the seed is derived from own cultivation. A major part of the gap between potential and actual yields is caused by biotic stresses such as pests and diseases (Lobell et al. 2009), which are expected to put an even larger pressure on yields with the predicted changes in climatic conditions (Chakrabortya and Newton 2011). The impact of breeding for resistance and developing pesticides to protect crops against these stresses is often limited and temporary, since pests and diseases evolve to break plant resistance or become resistant against pesticides (Normile 2008). Moreover, the use of fertilizers and pesticides needs to be reduced to safeguard the environment (Matson et al. 1997; Lansing and Kremer 2011). Therefore, improved farming practices and the utilization of ecological processes are crucial to improve rice yields. In the experiment reported here, selected methods from integrated pest management (IPM) (Glen et al. 1995; Gurr et al. 2004) and SRI (Uphoff 1999) were combined with integration of organic fertilizer application and incorporation of ducks and fish as productive components. It clearly demonstrated the potential of farming based on ecological processes in systems with increasing complexity for improvement of yields.

The selected SRI practices have a clear plant physiological basis. The larger spacing between plants allows for better development of individual plants and rooting systems due to reduced competition above and below ground (Kassam et al. 2011). Seeding in a nursery and transplanting 8–12 days after emergence results in more vigorous plant development in the field (Stoop 2011). Although synthetic fertilizers supply more nutrients to the crop that are often directly available, organic fertilizers such as compost can contribute to soil improvement and lift constraints on crop production related to the supply of micronutrients, in many cases more effectively than synthetic fertilizers (Kassam et al. 2011).

The experiment was conducted in a season with high cumulative precipitation amounts and large amounts per event when compared to the 20-year average. Although these conditions were very conducive for pest development, a provisional estimate of the total grain DM yield was high (10.6 Mg/ha) in the most complex treatment. Despite the fact that this amount was derived from up-scaling of yields from individual plant level and considerable caution is warranted, it indicates that the yields measured in this experiment were at least comparable to those of successful irrigated rice crops in field and on-farm experiments. In such trials, yields usually range between 5 and 7 Mg/ha for organic systems (Hariryoto 2011) and between 8 and 12 Mg/ha for conventional systems (Cassman and Pingali 1995). However, in the season of our experiment in the Pagelaran subdistrict the average conventional rice yields were 7.9 Mg/ha (CASM 2011).

The increase in yield along the complexity gradient indicated that the productivity in the more complex systems were robust to the high rainfall conditions probably due to beneficial effects of the synergies between system components. The effect of increasing system complexity on reducing the abundance of pest organisms and weeds (less frequent weeding in plots with ducks) was evident. In particular, the presence of ducks reduced the pest abundance (Fig. 2). Ducks eat insects and weeds. Fish can eat and uproot weeds, and can contribute to fungus suppression by feeding on mycelia (Xie et al. 2011). Moreover, the moving ducks and fish can hit rice stems resulting in an increased removal of dewdrops (and consequently less risk of spore generation and mycelium penetration) and in more insects falling into the water (Xie et al. 2011).

Nutrient cycling was probably enhanced by various processes, including the feeding and manure production by ducks and fish (Cagauan et al. 2000), the improved degradation of organic wastes due to improved aeration of the water by the animal movement (Bird et al. 2000), and the nitrogen fixation by the algae associated with the azolla fern. The activities of duck and fish such as trampling and stirring in the rice field also increase dissolved oxygen in the water. Higher dissolved oxygen lead methanogenic bacteria changes organic acid in the rice field to CO_2_ instead of CH_4_. Therefore, CH_4_ transfer from rice field to the air will be reduced (Zhiqiang et al. 2008). However, under aerobic conditions the nitrification process is also enhanced and as a consequence the production of N_2_O could be increased (Li et al. 2009).

Both the reduced pest and disease pressure and the improved nutrient status had a positive effect on yield, probably related to a more vigorous plant growth and better plant resistance. This was also reflected in morphological plant characteristics (higher leaf expansion rates) and improved plant physiological status (lower biomass DM content and specific leaf area). Here we have focused primarily on the beneficial effects on rice crop performance, but these effects are mutual, indicating true synergies. Both ducks and fish benefit from the presence of the rice plants that attract the insects that serve as feed. Also the azolla serves as a feed and fixes atmospheric nitrogen, and on the other hand benefits from the non-nitrogenous nutrients that are dissolved in the water and that originate from decomposition of compost and decayed plant material and from the excreta of ducks and fish (Cagauan et al. 2000). Moreover, the rice plants provide shade due to leaf expansion, a low-ammonia aquatic environment due to nutrient uptake, which are factors that positively influence fish (Xie et al. 2011).

The resulting gross margin was sufficient for smallholders to acquire the necessary inputs for the next season and to refund more than 70% of the initial credit. Alternatively, cooperation of rice producers with duck farmers can substantially reduce the required investments since ducklings and feed represent 74% of the extra costs and would also alleviate labor constraints, but this cooperation would depend on mutual commitment, social organization, and trust (Pretty and Smith 2004; Simmons and Birchall 2008). Similar positive economic benefits of ecologically based practices by smallholders have been observed for rice cultivation using IPM principles in Bangladesh (Dasgupta et al. 2007). Also integration of cocultures at field level into complex whole-farm systems could be feasible and profitable (e.g., Behera et al. 2008).

The successfulness of complex agro-ecosystems relying on ecological processes will depend on the system at hand and local conditions, but the approach to rice cultivation presented in this paper can serve as a suitable model system (cf., Lansing and Kremer 2011), since it consists of a diverse assemblage of water, plants, animals, and organic residues and because the influences of the various production factors could be clearly distinguished in our experiment. Although the revenues from the complex agro-ecosystems will be variable due to the strong dependence on ecological processes that are susceptible for environmental fluctuations (Morton 2007; Gregory et al. 2009), the improved nutrient status and biological weed and pest control enhance the robustness of the systems (Altieri 1999; Shennan 2008), as demonstrated in the extremely unfavorable conditions as experienced in the trial presented here. Farmers will require training to manage the timing and application rates of the various production factors. The initial investments that are required to add an increasing number of components in order to constitute complex agro-ecosystems that support ecological processes without relying on artificial inputs can contribute considerably to increasing rice grain yields and thus food security, and to improving the livelihood of resource-poor smallholders.

## Supporting Information

**Figure S1**. Changes in leaf and biomass characteristics at different days after transplanting of the rice plants.

**Figure S2**. Dry matter accumulation at different days after transplanting of the rice crop. R, rice; D, with ducks; C, with compost; F, with fish; A, with azolla.

**Figure S3**. The relation between total inputs and outputs of nutrients ([A] N = nitrogen, [B] P = phosphorus, [C] K = potassium) in systems without animals (R, RC, and RCA; blue symbols), with ducks (RD, RDC, RDCA; red symbols), and with ducks and fish (RDF, RDFC, and RDFCA; green symbols).

**Table S1**. Nutrient contents of inputs.

**Table S2**. Yield components of rice as affected by increasing complexity of the production systems due to adding ducks (D), compost (C), fish (F), and azolla (A).

**Table S3**. Uptake of nutrients (g m^−2^) by rice as affected by increasing complexity of the production systems due to adding ducks (D), compost (C), fish (F), and azolla (A).

**Table S4**. Details of financial calculations for treatments with rice with compost (RC) and including ducks, fish, compost, and azolla (RDFCA).
