# Supplementary material for: Complex agro-ecosystems for food security in a changing climate
Source: Ecol Evol. 2012 Jul;2(7):1696–704. doi: 10.1002/ece3.271 (PMC3434917; doi:10.1002/ece3.271)
Supplement: Supplementary file 1 [file ece30002-1696-SD1.doc]

Table S1. Nutrient contents of inputs.

| Name of input | Nitrogen (N)  content | Phosphorus (P)  content | Potassium (K)  content |
| --- | --- | --- | --- |
|  |  |  |  |
| Rice seed | 15.3 g kg^-1^ DM | 3.1 g kg^-1^ DM | 11.4 g kg^-1^ DM |
| Ducklings (30 g/duckling) | 33 g kg^-1^ | 1.6 g kg^-1^ | 2.0 g kg^-1^ |
| Juvenile fish (20 g/fish) | 32.5 g kg^-1^ | 1.7 g kg^-1^ | 3.2 g kg^-1^ |
| Azolla | 1.8 g kg^-1^ FM | 0.6 g kg^-1^ FM | 1.8 g kg^-1^ FM |
|  |  |  |  |
| Compost |  |  |  |
| Straw | 10.0 g kg^-1^ DM | 5.4 g kg^-1^ DM | 10.0 g kg^-1^ DM |
| Duck manure | 13.9 g kg^-1^ DM | 13.6 g kg^-1^ DM | 6.5 g kg^-1^ DM |
| Duckweed | 2.9 g kg^-1^ FM | 1.6 g kg^-1^ FM | 2.1 g kg^-1^ FM |
|  |  |  |  |
| Duck feed |  |  |  |
| Rice bran | 15.3 g kg^-1^ DM | 5.4 g kg^-1^ DM | 11.4 g kg^-1^ DM |
| Corn | 13.0 g kg^-1^ DM | 4.0 g kg^-1^ DM | 4.8 g kg^-1^ DM |
| Dried fish | 46.0 g kg^-1^ DM | 3.0 g kg^-1^ DM | 5.0 g kg^-1^ DM |

| 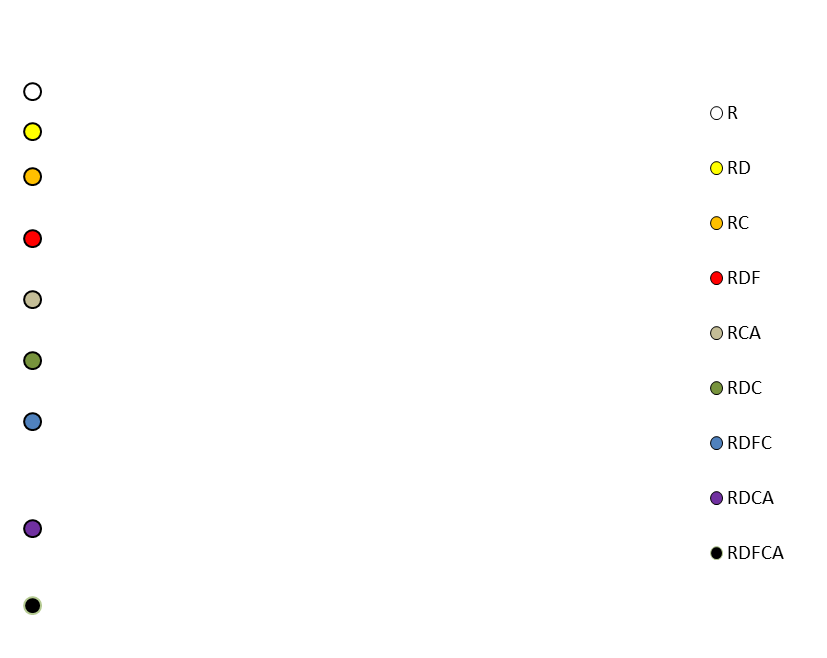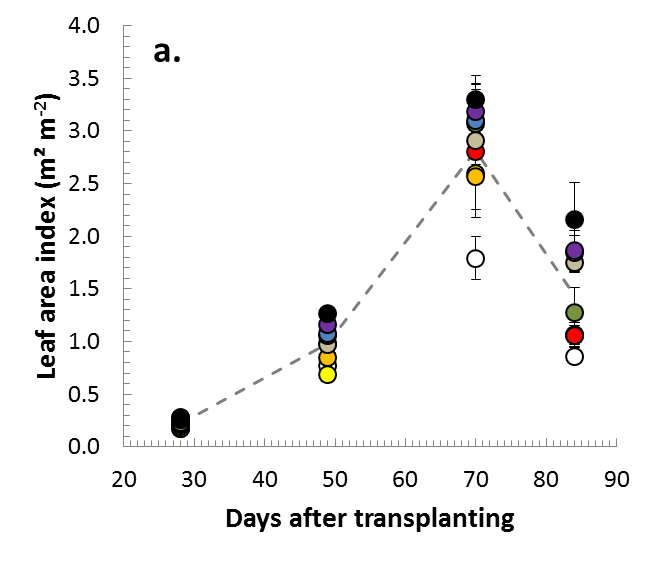 |  |
| --- | --- |
| 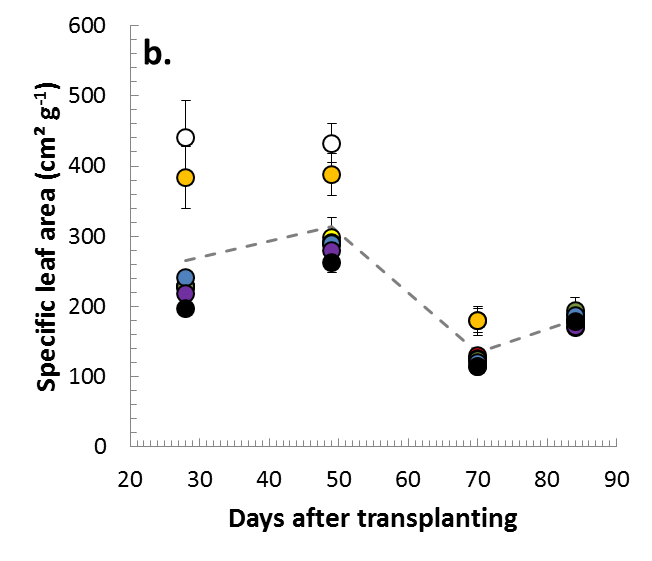 |
| 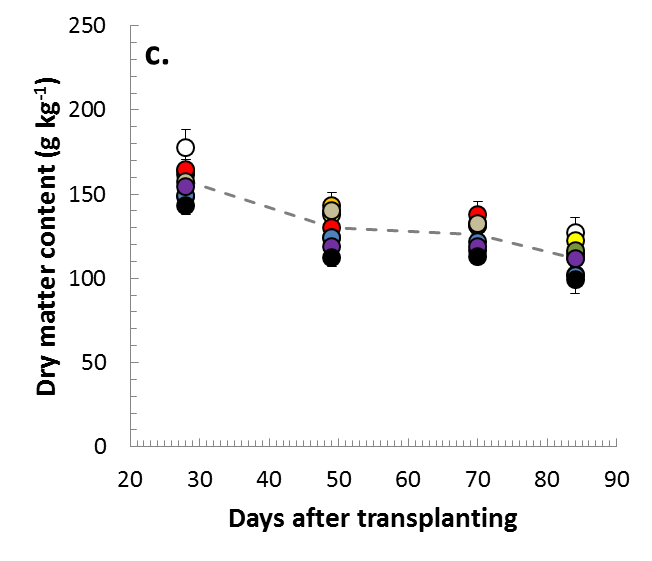 |
| Fig. S1. Changes in leaf and biomass characteristics at different days after transplanting of the rice plants. a. Leaf area index (m² leaf/ m² soil). b. Specific leaf area (cm² leaf/g leaf). c. Plant biomass dry matter content (g/kg). R = rice, D = with ducks, C = with compost, F = with fish, A = with azolla. The dashed line indicates the trend in the overall average. Error bars represent standard error of the mean (n=10). | |


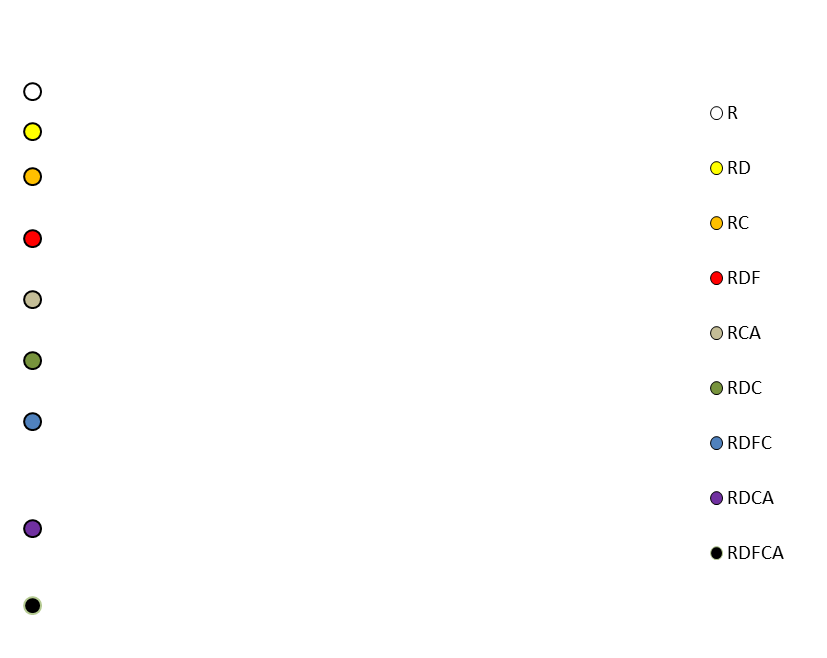


| 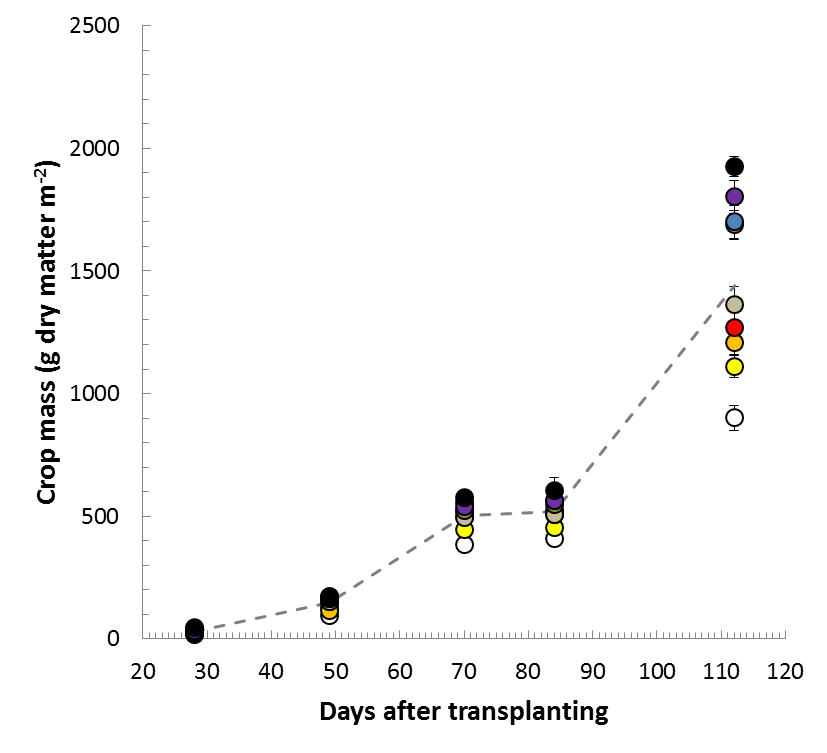 |  |
| --- | --- |
| Fig. S2. Dry matter accumulation at different days after transplanting of the rice crop. R = rice, D = with ducks, C = with compost, F = with fish, A = with azolla. The dashed line indicates the trend in the overall average. Error bars represent standard error of the mean (n=10). | |

Table S2. Yield components of rice as affected by increasing complexity of the production systems due to adding ducks (D), compost (C), fish (F) and azolla (A). Means with different letters are significantly different, following the Tukey’s post hoc test. n=10 per treatment.

| Variable | Pest  management |  | Nutrient management | | | Mean |
| --- | --- | --- | --- | --- | --- | --- |
|  |  |  | R | +C | +C+A |  |
|  |  |  |  |  |  |  |
| Number of | R |  | 13 | 16 | 17 | 15a |
| panicles per | +D |  | 17 | 23 | 22 | 20b |
| hill | +D+F |  | 18 | 23 | 23 | 21b |
|  |  |  |  |  |  |  |
|  | Mean |  | 16a | 20b | 21b |  |
|  |  |  |  |  |  |  |
| Number of | R |  | 108 | 123 | 141 | 124a |
| grains per | +D |  | 101 | 142 | 159 | 134b |
| panicle | +D+F |  | 114 | 139 | 158 | 137b |
|  |  |  |  |  |  |  |
|  | Mean |  | 108a | 135b | 153c |  |
|  |  |  |  |  |  |  |
| 1000-grain | R |  | 26 | 26 | 27 | 26a |
| weight (g) | +D |  | 25 | 27 | 27 | 27b |
|  | +D+F |  | 26 | 27 | 28 | 27b |
|  |  |  |  |  |  |  |
|  | Mean |  | 26a | 27b | 27b |  |
|  |  |  |  |  |  |  |

Table S3. Uptake of nutrients (g m^-2^) by rice as affected by increasing complexity of the production systems due to adding ducks (D), compost (C), fish (F) and azolla (A). Means with different letters are significantly different, following the Tukey’s post hoc test. n=10 per treatment.

| Variable | Pest  management |  | Nutrient management | | | Mean |
| --- | --- | --- | --- | --- | --- | --- |
|  |  |  | R | +C | +C+A |  |
|  |  |  |  |  |  |  |
| Nitrogen | R |  | 9.4 | 14.5 | 17.5 | 13.7a |
| (N) | +D |  | 15.5 | 22.3 | 25.1 | 20.9b |
|  | +D+F |  | 18.6 | 24.2 | 30.0 | 24.2c |
|  |  |  |  |  |  |  |
|  | Mean |  | 14.5a | 20.3b | 24.1c |  |
|  |  |  |  |  |  |  |
| Phosphorus | R |  | 2.9 | 4.5 | 6.1 | 4.5a |
| (P) | +D |  | 3.9 | 6.2 | 9.7 | 6.6b |
|  | +D+F |  | 5.1 | 5.7 | 10.2 | 6.9b |
|  |  |  |  |  |  |  |
|  | Mean |  | 3.9a | 5.5b | 8.7c |  |
|  |  |  |  |  |  |  |
| Potassium | R |  | 5.4 | 9.0 | 12.6 | 9.0a |
| (K) | +D |  | 10.9 | 16.5 | 18.0 | 15.1b |
|  | +D+F |  | 12.3 | 17.0 | 21.9 | 17.0c |
|  |  |  |  |  |  |  |
|  | Mean |  | 9.5a | 14.1b | 17.5c |  |
|  |  |  |  |  |  |  |

| 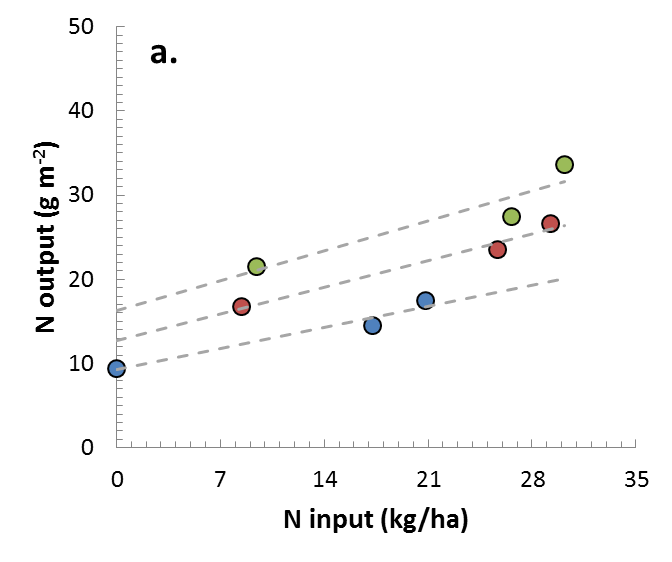 |
| --- |
| 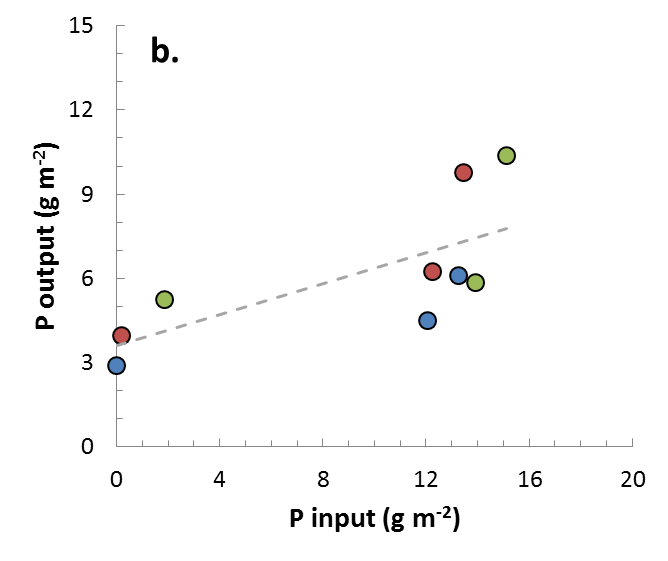 |
| 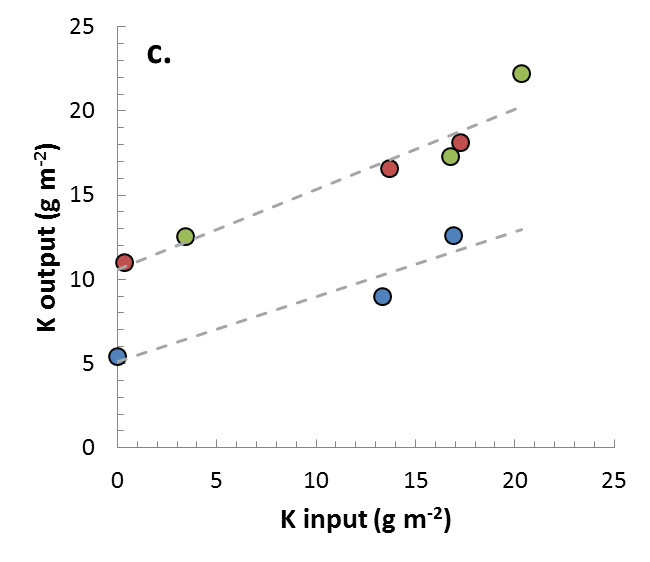 |
| Fig. S3. The relation between total inputs and outputs of nutrients (a. N = nitrogen, b. P = phosphorus, c. K = potassium) in systems without animals (R, RC and RCA; blue symbols), with ducks (RD, RDC, RDCA; red symbols) and with ducks and fish (RDF, RDFC and RDFCA; green symbols). |

Table S4. Details of financial calculations for treatments with rice with compost (RC) and including ducks, fish, compost and azolla (RDFCA).

| **Attribute** | **Units (per ha)** | **Unit price** | **R** | **RDFCA** |
| --- | --- | --- | --- | --- |
|  |  |  |  |  |
| **COSTS** |  |  |  |  |
| Duck manure (sacks) | 300 | 0.46 | 139.37 | 139.37 |
| Rice seeds (kg) | 20 | 0.87 | 17.42 | 17.42 |
| Ducklings | 400 | 1.16 |  | 464.58 |
| Nile tilapia, 10 cm | 5000 | 0.03 |  | 174.22 |
| Duck feed (kg) | 4111 | 0.23 |  | 954.94 |
| Azolla | 1 | 30.20 |  | 30.20 |
| Bio-pesticides | 1 | 1.16 | 1.16 | 1.16 |
| Renting tools |  |  |  |  |
| - Plow | 1 | 174.22 | 174.22 | 174.22 |
| - Harvest equipment | 1 | 27.87 | 27.87 | 27.87 |
| Labor (hours) |  |  |  |  |
| - Composting | 20 | 4.07 | 81.30 | 81.30 |
| - Soil preparation | 4 | 4.07 | 16.26 | 16.26 |
| - Seeding and transplanting | 22 | 4.07 | 89.43 | 89.43 |
| - Pest management | 1 | 4.07 | 4.07 | 4.07 |
| - Weed management | 24 | 4.07 | 97.56 |  |
|  | 18 | 4.07 |  | 73.17 |
| - Water management | 1 | 17.42 | 17.42 | 17.42 |
| - Duck management | 6 | 52.26 |  | 313.59 |
| - Fish management | 15 | 4.07 |  | 60.98 |
| **Total costs** |  |  | **666.09** | **2640.19** |
|  |  |  |  |  |
| **REVENUES** |  |  |  |  |
| Rice (kg) | 5600 | 0.35 | 1951.22 |  |
| Rice (kg) | 10500 | 0.35 |  | 3658.54 |
| Fish (kg) | 625 | 0.81 |  | 508.13 |
| Ducks | 320 | 3.48 |  | 1114.98 |
| **Total revenues** |  |  | **1951.22** | **5281.65** |
|  |  |  |  |  |
| **Gross margin** |  |  | **1285.13** | **2641.46** |
